# A Novel Three-Gene Model Predicts Prognosis and Therapeutic Sensitivity in Esophageal Squamous Cell Carcinoma

**DOI:** 10.1155/2019/9828637

**Published:** 2019-11-25

**Authors:** Fa-Min Zeng, Jian-Zhong He, Shao-Hong Wang, De-kai Liu, Xiu-E. Xu, Jian-Yi Wu, En-Min Li, Li-Yan Xu

**Affiliations:** ^1^The Key Laboratory of Molecular Biology for High Cancer Incidence Coastal Chaoshan Area, Shantou University Medical College, Shantou, Guangdong, China; ^2^Department of Biochemistry and Molecular Biology, Shantou University Medical College, Shantou, Guangdong, China; ^3^Guangdong Provincial Engineering Research Center of Molecular Imaging, The Fifth Affiliated Hospital, Sun Yat-sen University, Zhuhai, Guangdong, China; ^4^Department of Pathology, The Fifth Affiliated Hospital, Sun Yat-sen University, Zhuhai, Guangdong, China; ^5^Department of Pathology, Shantou Central Hospital, Affiliated Shantou Hospital of Sun Yat-sen University, Shantou, Guangdong, China; ^6^Department of Medical Records Management, Shenzhen People's Hospital, Shenzhen, Guangdong, China

## Abstract

To precisely predict the clinical outcome and determine the optimal treatment options for patients with esophageal squamous cell carcinoma (ESCC) remains challenging. Prognostic models based on multiple molecular markers of tumors have been shown to have superiority over the use of single biomarkers. Our previous studies have identified the crucial role of ezrin in ESCC progression, which prompted us to hypothesize that ezrin-associated proteins contribute to the pathobiology of ESCC. Herein, we explored the clinical value of a molecular model constructed based on ezrin-associated proteins in ESCC patients. We revealed that the ezrin-associated proteins (MYC, PDIA3, and ITGA5B1) correlated with the overall survival (OS) and disease-free survival (DFS) of patients with ESCC. High expression of MYC was associated with advanced pTNM-stage (*P*=0.011), and PDIA3 and ITGA5B1 were correlated with both lymph node metastasis (PDIA3: *P* < 0.001; ITGA5B1: *P*=0.001) and pTNM-stage (PDIA3: *P*=0.001; ITGA5B1: *P*=0.009). Furthermore, we found that, compared with the current TNM staging system, the molecular model elicited from the expression of MYC, PDIA3, and ITGA5B1 shows higher accuracy in predicting OS (*P* < 0.001) or DFS (*P* < 0.001) in ESCC patients. Moreover, ROC and regression analysis demonstrated that this model was an independent predictor for OS and DFS, which could also help determine a subgroup of ESCC patients that may benefit from chemoradiotherapy. In conclusion, our study has identified a novel molecular prognosis model, which may serve as a complement for current clinical risk stratification approaches and provide potential therapeutic targets for ESCC treatment.

## 1. Introduction

Esophageal cancer is the sixth leading cause of cancer-related deaths and the eighth most common type of malignant gastrointestinal cancer in the world [1, 2]. Adenocarcinoma and squamous cell carcinoma (ESCC) are the two major types of esophageal cancer, with the latter accounting for the 90% of cases worldwide [3]. In China, ESCC still remains the highest incidence and cancer-induced mortality rates, and the long-term prognosis of patients with ESCC is less than 20%, despite improvements in treatments such as surgical resection and adjuvant chemoradiation [4, 5]. This poor prognosis for ESCC patients is highly associated with the difficult nature of diagnosing early-stage ESCC and the frequent occurrence of local invasion and distant metastasis [5]. In addition, conventional chemotherapy and radiotherapy treatments are relatively ineffective [6]. Therefore, seeking novel molecular prognostic markers that can help identify patients at high risk and improving their prognosis are urgent needs in the clinic.

However, signal molecular marker cannot meet the clinical requirements for biomarkers, such as high sensitivity and specificity, and it is more accurate than the current clinical staging system [7]. In the last few years, studies have demonstrated that combinations of multiple biomarkers were more sensitive and reliable than single molecular marker. Although several prognostic biomarkers for ESCC have been reported [8–12], there is still no ideal biomarker for clinical use.

Ezrin as a member of the ezrin/radixin/moesin (ERM) protein family plays an important role in regulating the growth and metastatic of cancer [13, 14]. In our previous studies, we showed that ezrin was upregulated in ESCC and promoted cellular proliferation and invasiveness of ESCC cells [15]. Furthermore, Ezrin might be a new prognostic molecular marker for ESCC patients [16]. Ezrin was also known as a key molecule connected with many other molecules in the biology of tumor development [17]. In these ezrin-related proteins, our previous studies identified that three proteins, i.e., MYC, PDIA3, and ITGA5B1, correlated with patients' survival [11, 12]. MYC, a protooncogene, plays an integral role in a variety of normal cellular functions [18]. MYC amplification is a recurrent event in many tumors and contributes to tumor development and progression [19–22]. The progress of MYC-induced tumorigenesis in prostate cancer cells entails MYC binding to the ezrin gene promoter and the induction of its transcription [23]. Meanwhile, the induction of ezrin expression is essential for MYC-stimulated invasion [23]. PDIA3 (protein disulfide isomerase family A, member 3), also known as ERp57, is one of the main members of the protein disulfide isomerase (PDI) gene family and is identified primarily as enzymatic chaperones for reconstructing misfolded proteins within the endoplasmic reticulum (ER) [24]. Several studies have linked PDIA3 to different types of cancer, including breast [25], ovarian [26], and colon [27] cancers. In ESCC, we found that PDIA3 interacted with ezrin, and it was not only involved in the development and progression of ESCC but also related to OS and DFS of ESCC patients [12]. ITGA5B1 is a member of the integrin family which plays a significant role in cell adhesion to the extracellular matrix (ECM) [28, 29]. In ESCC, ITGA5B1 upregulates the expression of ezrin through the L1CAM [30].

Although ezrin plays a pivotal role in ESCC progression, the clinical significance of ezrin-related proteins (MYC, PDIA3, and ITGA5B1) has not been thoroughly investigated in ESCC patients. Clinicopathological analyses of these ezrin-interacting proteins may further our understanding of the function of ezrin and provide therapeutic targets for ESCC. In the current study, we found that a three-gene signature comprised of MYC, PDIA3, and ITGA5B1 could independently predict ESCC patient survival.

## 2. Materials and Methods

### 2.1. Patients and Specimens

For this retrospective study, 284 cases of formalin-fixed, paraffin-embedded ESCC tissue were collected from the Shantou Central Hospital between November 2007 and January 2010. All patients underwent curative resection and were confirmed as having ESCC by pathologists in the Clinical Pathology Department of the Hospital. Information on age, gender, and histopathological factors was obtained from the medical records and shown in Table 1. An independent validation set (GSE53622 and GSE5364) was obtained from the publicly available GEO database (https://www.ncbi.nlm.nih.gov/). We excluded the ESCC patients without clinical survival information, and the clinicopathological information was shown in [Supplementary-material supplementary-material-1]. Overall survival (OS) was defined as the interval between surgery and death from tumors or between surgery and the last observation taken for surviving patients. Disease-free survival (DFS) was defined as the interval between surgery and diagnosis of relapse or death. Ethical approval was obtained from the ethical committee of the Central Hospital of Shantou City and the ethical committee of the Medical College of Shantou University, and only resected samples from surgical patients giving written informed consent were included for use in research.

### 2.2. Tissue Microarrays (TMAs) and Immunohistochemistry (IHC)

TMAs were constructed based on standard techniques as previously described [12]. IHC was performed using the PV-9000 2-step Polymer Detection System (ZSGB-BIO, Beijing, China) and Liquid DAB Substrate Kit (Invitrogen, San Francisco, CA) according to the manufacturer's instructions and has been described in our previous studies [12]. The primary mouse monoclonal MYC antibody (1 : 100 dilution; Santa Cruz Biotechnology, USA), anti-PDIA3 antibody (polyclonal, 1 : 700 dilution; sigma, Saint Louis, MO), and anti-ITGA5B1 antibody (monoclonal, 1 : 50 dilution; millipore, USA) were used in this study.

### 2.3. Evaluation of IHC Variables

The protein expression was evaluated by an automated quantitative pathology imaging system (PerkinElmer, Waltham, MA, USA), as described previously [11]. Briefly, as shown in [Supplementary-material supplementary-material-1], the automated image acquisition and color images were obtained using Vectra 2.0.8 software. Subsequently, the spectral libraries were constructed using Nuance 3.0 software. And then, the color images were evaluated by Inform 1.2 software as follows: (1) segmentation of the tumor region from the tissue compartments, (2) segmentation of the tumor region from the tumor region, and (3) H score calculation (=(% at 0) *∗* 0 + (% at 1+) *∗* 1 + (% at 2+) *∗* 2 + (% at 3+) *∗* 3) based on the optical density which produces a continuous protein expression value in the range of 0 to 300.

### 2.4. Construction of a Survival Predictive Model

Firstly, we used a univariate Cox proportional hazards regression analysis to evaluate the correlation between survival and each protein. Subsequently, we constructed a predictive model by the summation of the expression of each biomarker (high = 1, low = 0) multiplied by its regression coefficient, as described in the following equation: *Y* = (*β*1) × MYC + (*β*2) × PDIA3 + (*β*3) × ITGA5B1 [9]. Patients were then divided into three groups (high-risk, medium-risk, and low-risk) by the cut-off value generated by X-tile software [31].

### 2.5. Statistical Analysis

The SPSS v19.0 program was used for statistical analysis. Cumulative survival time was calculated by the Kaplan-Meier (K-M) method and analyzed by the log-rank test. The association of biomarkers and clinicopathological factors was evaluated by Fisher's exact test. The Cox proportional hazards regression model was used for univariate and multivariate analyses. The predictive value of the parameters was determined by receiver operating characteristic (ROC) curve analysis. *P* < 0.05 was considered to be statistically significant.

## 3. Results

### 3.1. Immunohistochemical Characteristics of 3 Biomarkers

**Figure 1:**
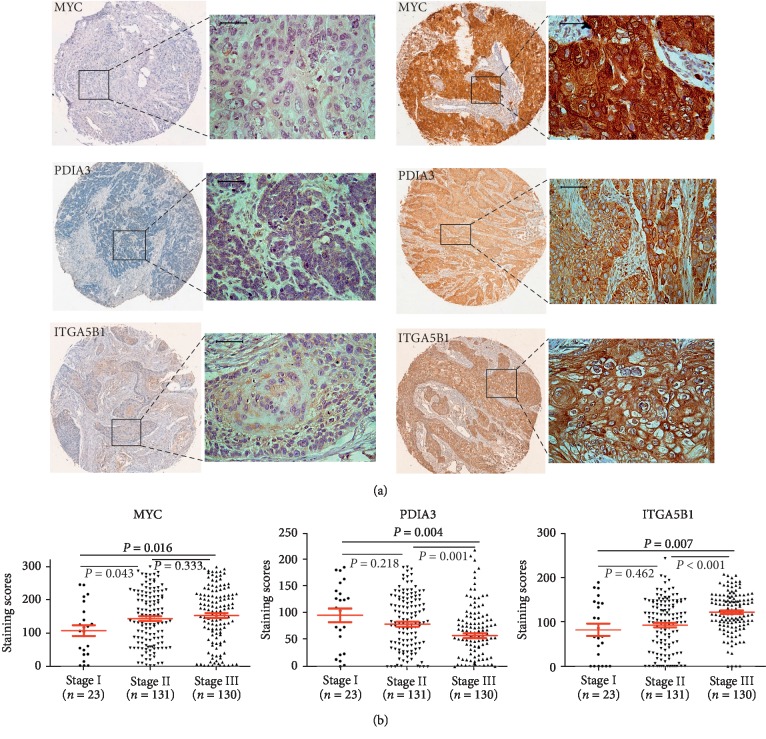
Expression of MYC, PDIA3, and ITGA5B1 in ESCC. (a) Representative images of IHC staining for MYC, PDIA3, and ITGA5B1 in ESCC samples (scale bars = 50 *μ*m). (b) The H scores of MYC, PDIA3, and ITGA5B in different clinical stages (stages I, II, and III) of ESCC were shown by scatter diagram (*P* < 0.05, independent-samples *t*-test).

The expression levels of MYC, PDIA3, and ITGA5B1 protein in ESCC were examined by IHC. As shown in Figure 1(a), MYC, PDIA3, and ITGA5B1 were mainly localized in the cytoplasm. We further investigated the association between the expression of these 3 biomarkers and clinicopathological parameters. There was no significant correlation between the 3 markers and age, gender, tumor size, histologic grade, or invasive depth, etc. Nonetheless, low-expression of PDIA3 or high expression of ITGA5B1 significantly correlated with lymph node (LN) metastasis, whereas no correlation was found between MYC and LN metastasis (Table 2). In addition, PDIA3 had a negative correlation while MYC and ITGA5B1 had a positive correlation with pTNM-stage (Table 2). In support of these correlation analyses, MYC and ITGA5B1 showed increased expression in tumors with high clinical stage; in contrast, PDIA3 expression was downregulated in stage III tumors compared with those with stages I and II (Figure 1(b)).

### 3.2. Prognostic Significance of MYC, PDIA3, and ITGA5B1 in Patients with ESCC

**Figure 2:**
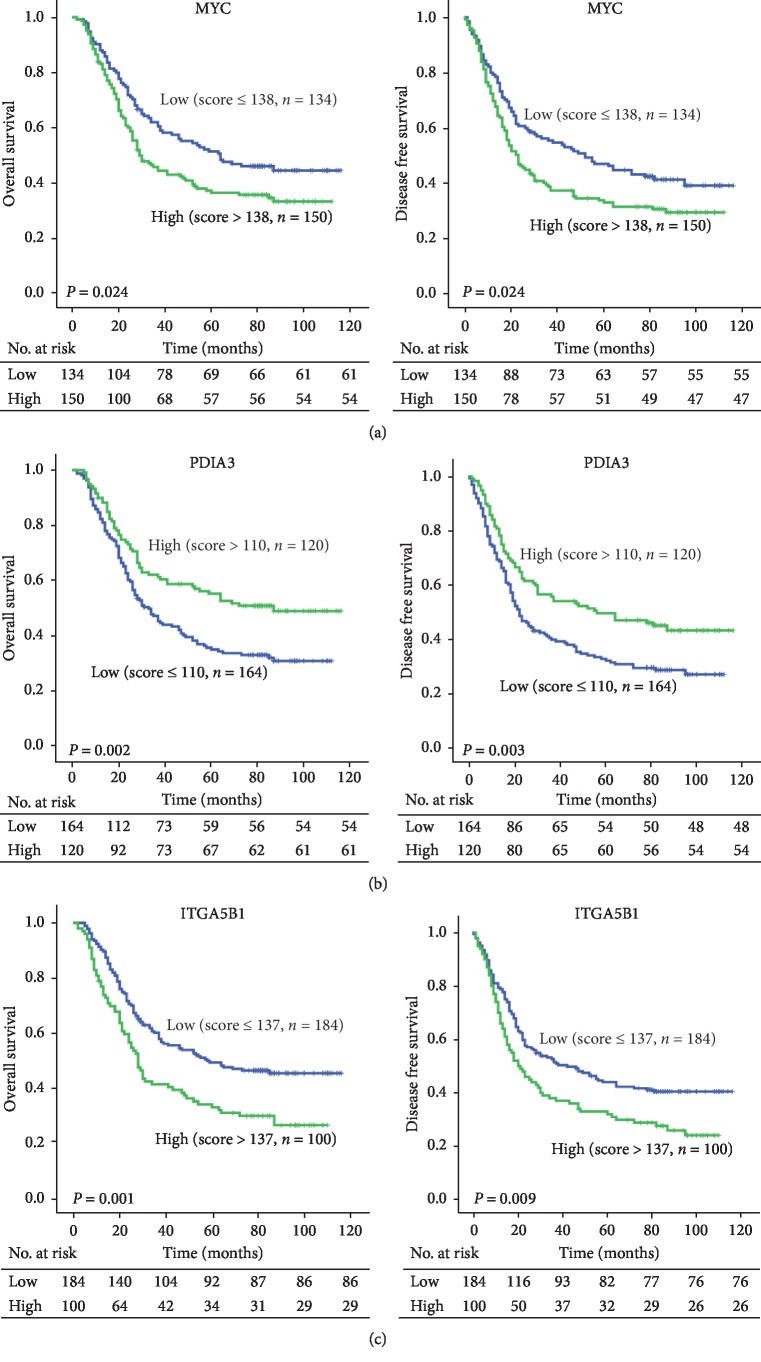
K-M survival analysis in ESCC patients based on the expression of MYC, PDIA3, and ITGA5B1. The H scores of each protein were divided into low and high groups as determined by X-tile, and the number of patients who were at risk at specific times was labeled under the *x*-axis (*P* < 0.05, log-rank test).

To further explore the clinical significance of MYC, PDIA3, and ITGA5B1 in ESCC patients, Kaplan-Meier analysis and log-rank test were performed. As shown in Figure 2, high expression of MYC or ITGA5B1 was significantly associated with poor prognosis (MYC: OS, *P*=0.024, DFS, *P*=0.024; ITGA5B1: OS, *P*=0.001, DFS, *P*=0.009, Figures 2(a) and 2(c)). However, the overexpression of PDIA3 trended to predict a favorable OS (*P*=0.002) and DFS (*P*=0.003, Figure 2(b)). Besides, because ITGA5B1 is a heterodimer of alpha and beta subunit, we used the expression level of ITGA5 instead of ITGA5B1 in microarray data, and the predictive value of MYC, PDIA3, and ITGA5 was further validated in an independent cohort (GSE53622 and GSE5364).

The results for validation set were in line with those in generation set (Supplementary [Supplementary-material supplementary-material-1]). Univariate Cox regression analysis further identified that these 3 molecules were significantly associated with OS (MYC: *P*=0.026; PDIA3: *P*=0.003; ITGA5B1: *P*=0.001) and DFS (MYC: *P*=0.026; PDIA3: *P*=0.004; ITGA5B1: *P*=0.010, Table 3).

### 3.3. A Molecular Prognostic Model of the 3 Biomarkers Signature

**Figure 3:**
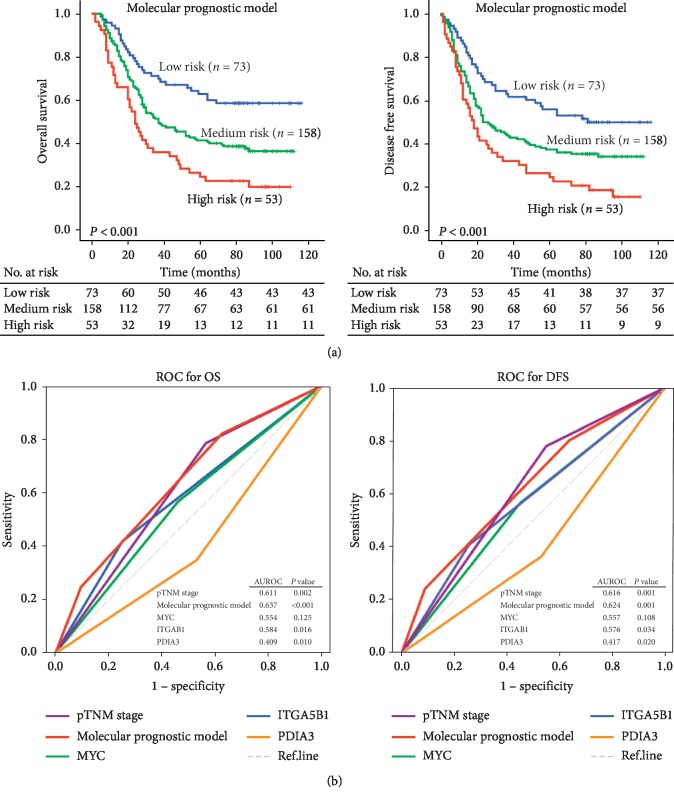
Predictive value of the molecular model. (a) K-M survival curves showing that the OS and DFS had a striking contrast between the ESCC patients in low-, medium-, and high-risk groups. (b) Receiver operating characteristic (ROC) curve was used to evaluate the ability of the molecular model for OS or DFS compared with each biomarker alone or the pTMN-stage.

We then evaluated the prognostic value of a molecular model that takes consideration of all the 3 biomarkers. To this end, we calculated the risk score *Y* = (*β*1) *∗* (MYC) + (*β*2) *∗* (PDIA3) + (*β*3) *∗* (ITGA5B1). In this dataset, the regression coefficients (*β*1 = 0.347, *β*2 = −0.482, *β*3 = 0.501) were calculated by univariate Cox proportional hazards analysis. All patients were divided into low-, medium-, and high-risk groups based on the *Y* scores, and the optimal cut-off values were determined by the X-tile software based on patients' prognosis [31]. Kaplan–Meier analysis further demonstrated that patients in the low-risk group indeed had markedly prolonged survival (OS: *P* < 0.001: DFS: *P* < 0.001, Figure 3(a)). The 5-year OS for low-, medium-, and high-risk groups was 62.9%, 41.3%, and 24.5%, respectively. Similar results were obtained for 5-year DFS in those groups, which were 56.0%, 37.4%, and 24.5%, respectively (Figure 3(a)). To validate whether this molecular prognostic model can serve as an independent predictor for OS and DFS, we carried out both univariate and multivariate analyses. As shown in Table 3, our newly defined molecular prognostic model, along with pTNM-stage and tumor size, was independent prognostic factors (Table 3). Moreover, receiver operating characteristic (ROC) analysis indicated that the predictive power of this molecular prognostic model was higher compared to each biomarker individually or the pTNM-stage (Figure 3(b)). The predictive value and power of molecular model for OS also yielded similar results from validation set as shown in [Supplementary-material supplementary-material-1].

### 3.4. The Potential of the Molecular Prognostic Model in Identifying ESCC Patients Who Can Benefit from Chemoradiotherapy

**Figure 4:**
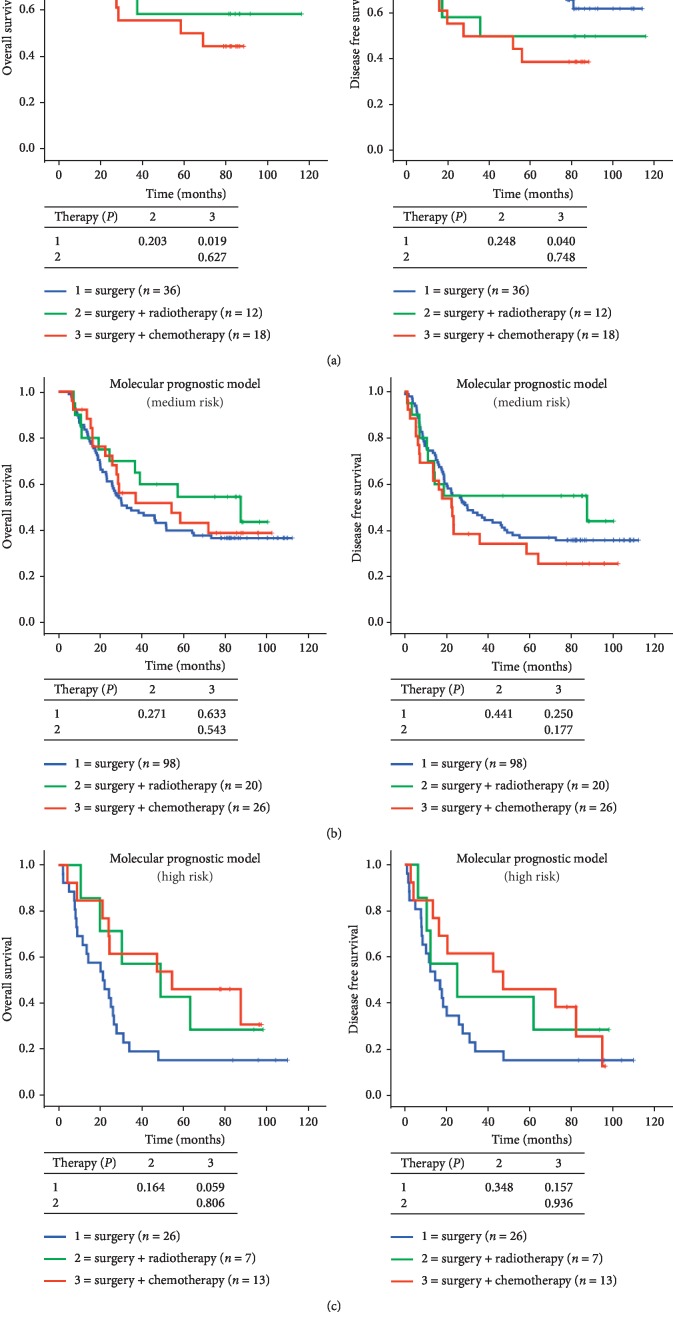
K-M survival curves indicate that radiotherapy and chemotherapy prolonged patients' OS and DFS in high-risk group but shortened patients' survival in low-risk group.

As shown in Table 1, chemoradiotherapy did not markedly prolong the OS and DFS of ESCC patients. To test the utility of the molecular prognostic model for predicting therapeutic efficacy, we performed K-M survival analysis. Our results showed that the OS and DFS of patients who were treated with surgery only were higher compared with those who received surgery + radiotherapy or surgery + chemotherapy in the low-risk group (Figure 4(a)). However, the opposite was true for patients in the high-risk group, in which ESCC patients who received only surgery had an unfavorable outcome (Figure 4(c)). Radiotherapy and chemotherapy tended to prolong patients' survival as the risk went up as determined by our molecular prognostic model. In particular, patients treated with surgery + chemotherapy in the high-risk group had the most favorable OS and DFS compared with surgery alone and surgery + radiotherapy (Figure 4).

## 4. Discussion

ESCC is one of the most prevalent and lethal cancers in Asian [4]; however, there is no effective molecular signatures for predicting the effectiveness of adjuvant treatments and prognosis in the clinic. Previous studies demonstrated that the cytoskeleton changes are intimately associated with cancer invasion and metastasis [32]. In support of this notion, our research has confirmed that the membrane-cytoskeletal linking protein ezrin contributes significantly to ESCC progression [15]. In this study, we attempted to generate an effective molecular model based on ezrin-related proteins (MYC, PDIA3, and ITGA5B1) for potential clinical applications. Our data highlight that a molecular model elicited from MYC, PDIA3, and ITGA5B1 has superior prognostic values compared with pTNM-stage, which also facilitates the identification of ESCC patients who may benefit from chemoradiotherapy.

Ezrin, a membrane-cytoskeleton linker, plays a major role in promoting tumor progression [23, 33]. Our previous study has identified the mislocalization of ezrin during ESCC development, in which membranous ezrin in normal epithelial cells becomes cytoplasmic in ESCC [34]. This abnormal localization changes the interacting proteins of ezrin, which has been shown to be critical for regulating tumor cell survival, invasion, and metastasis [12, 17]. The expressions of MYC, PDIA3, and ITGA5B1 have been demonstrated to play critical roles in various malignant tumors and are independent prognostic factors in certain cancers [12, 35, 36].

It is important to note that although ESCC patients with higher risk predicted by our three-protein molecular model had poor prognosis, these patients might benefit from adjuvant therapies such as chemoradiotherapy, which improved their survival compared with surgical treatment alone. Compared with the model using three different genes (PPARG, MDM2, and NANOG), which we reported in 2015 [9], the current molecular model not only accurately predicts the OS of patients with ESCC but also predicts the DFS and sensitivity to chemoradiation. This makes it much more practical for clinical application. Our results are in line with other clinical studies, which have shown that high expression and rearrangement of MYC are associated with better response to chemoradiotherapy compared with patients without these abnormalities [37, 38]. The mechanism behind this observation is probably related to the biological function of MYC in promoting DNA replication and cell cycle distribution [39]. As chemoradiotherapy utilizes the effects of DNA damage-induced cytotoxicity in neoplastic cells, it is not surprising to see an association between MYC and chemo/radiosensitivity in ESCC patients. Indeed, overexpression of MYC has been shown to render tumor cells susceptible to chemotherapeutics, such as etoposide, doxorubicin, and camptothecin [40]. Nevertheless, MYC remains an attractive molecular target for therapy due to its high oncogenic properties [41]. Antisense oligonucleotides (ASOs) targeting MYC have been shown to block cell proliferation and induce apoptosis in solid and hematologic tumors [41, 42].

Compared with MYC, relatively little is known about the biological function of ITGA5B1 in carcinoma. Recent studies suggest that ITGA5B1 can prevent cell anoikis through suppressing inflammation- and oxidative stress-related genes [43, 44]. ITGA5B1 is especially more noticeable in regulating cell adhesion [45], and it can promote early peritoneal metastasis in serous ovarian cancer [46]. In line with the protumorigenic role of ITGA5B1, we are the first to uncover the high expression of this protein in more advanced and metastatic ESCC tumors with unfavorable prognosis. Further studies are needed to delineate the mechanisms behind the deregulation of ITGA5B1 and its biological function in ESCC. PDIA3 has been shown to confer chemo/radioresistance to various types of tumor cells such as ovarian carcinoma [47, 48]. PDIA3 expression level is correlated with the clinical outcome of patients with ovarian carcinoma who receive chemoradiotherapy, and the sensitivity to paclitaxel can be enhanced by PDIA3 silencing [47, 48]. In ESCC, we found that PDIA3 decreased gradually with the progress of stage and related to favorable prognosis, which was in accord with the findings in gastric cancer [49], but contrary to those in hepatocellular carcinoma [50]. The favorable prognostic value of PDIA3 in ESCC implies that ESCC patients with high expression of PDIA3 may be more sensitive to chemotherapy such as paclitaxel, but further studies are warranted. These contrasting observations can be attributed to the differences in the carcinogenic machinery between ESCC and other carcinomas.

Taken together, these data suggest that MYC, PDIA3, and ITGA5B1 may serve as potential therapeutic targets for ESCC treatment, and cotargeting of these biomarkers might be more effective than targeting a single biomarker alone. Importantly, this study provides a clinically applicable molecular model that can more precisely predict clinical outcome than pTNM-stage, which may also facilitate the identification of ESCC patients who can benefit from radiotherapy or chemotherapy.

## Figures and Tables

**Table 1:** The clinicopathological characteristics of generation dataset of patients with ESCC.

Clinical and pathological indexes	Case no.	5-year OS (%)	*P* ^*∗*^	5-year DFS (%)	*P* ^*∗*^
Specimens	284				
Mean age	58.7				
Age (year)					
≤58	148	48.1	0.036	43.4	0.207
>58	136	39.1		35.8	

Gender					
Male	220	44.8	0.387	40.5	0.915
Female	64	40.2		37.2	

Therapies					
Only surgery	160	45.2	0.080	42.0	0.070
Surgery + radiotherapy	39	53.6		51.3	
Surgery + chemotherapy	57	46.2		36.4	
Surgery + chemoradiotherapy	28	17.9		17.9	

Tumor size					
≤3 cm	67	55.6	0.057	54.4	0.021
3–5 cm	134	43.5		37.9	
>5 cm	83	34.7		31.1	

Tumor location					
Upper	16	33.5	0.463	25.0	0.127
Middle	122	45.6		44.8	
Lower	146	43.3		37.2	

Histologic grade					
G1	45	57.7	0.001	57.7	<0.001
G2	219	43.5		38.3	
G3	20	15.0		15.0	

Invasive depth					
T1	13	84.6	0.005	84.6	0.013
T2	42	50.0		45.2	
T3	229	40.2		36.2	

Lymph node metastasis					
N0	141	58.1	<0.001	53.5	<0.001
N1	81	44.0		39.0	
N2	46	15.2		13.0	
N3	16	0.0		0.0	

pTNM-stage					
I	23	82.6	<0.001	82.6	<0.001
II	131	54.2		49.2	
III	130	26.4		22.6	

^*∗*^Log-rank test of Kaplan–Meier method; *P* < 0.05 was considered significant. All patients underwent surgical treatment. OS: overall survival. DFS: disease-free survival.

**Table 2:** The correlation between 3 markers and clinicopathological characteristics in ESCC.

Variables	MYC^a^		*P* ^*∗*^	PDIA3^b^		*P* ^*∗*^	ITCA5B1^c^		*P* ^*∗*^
	Low	High		Low	High		Low	High	
Age (year)									
≤58	67	81	0.425	84	64	0.725	92	56	0.334
>58	68	68		80	56		92	44	

Gender									
Male	109	111	0.208	127	93	0.990	137	83	0.100
Female	26	38		37	27		47	17	

Therapies									
Only surgery	85	75	0.067	97	63	0.588	107	53	0.849
Surgery + radiotherapy	14	25		20	19		25	14	
Surgery + chemotherapy	21	36		30	27		35	22	
Surgery + radiochemotherapy	15	13		17	11		17	11	

Tumor size									
≤3 cm	39	28	0.101	41	26	0.303	43	24	0.489
3–5 cm	62	72		71	63		83	51	
>5 cm	34	49		52	31		58	25	

Tumor location									
Upper	6	10	0.307	9	7	0.383	8	8	0.395
Middle	64	58		65	57		82	40	
Lower	65	81		90	56		94	52	

Histologic grade									
G1	25	20	0.499	20	25	0.054	32	13	0.588
G2	101	118		129	90		140	79	
G3	9	11		15	5		12	8	

Invasive depth									
T1 + T2	32	23	0.078	37	18	0.111	34	21	0.607
T3 + T4	103	126		127	102		150	79	

Lymph node metastasis									
N0	73	68	0.156	64	77	<0.001	105	36	0.001
N1 + N2 + N3	62	81		100	43		79	64	

pTNM-stage									
I	17	6	0.011	10	13	0.001	16	7	0.009
II	65	66		64	67		96	35	
III	53	77		90	40		72	58	

^*∗*^Fisher's exact test. *P* value < 0.05 was considered significant.

**Table 3:** Univariate and multivariate analyses of factors associated with overall survival (OS) and disease-free survival (DFS) in patients with ESCC.

Variables	Univariate analysis				Multivariate analysis			
	OS		DFS		OS		DFS	
	HR (95% CI)	*P*	HR (95% CI)	*P*	HR (95% CI)	*P*	HR (95% CI)	*P*
Age (>58 vs. ≤58)	1.376 (1.017 to 1.861)	0.039	1.203 (0.900 to 1.609)	0.213	1.498 (1.082 to 2.073)	0.015		
Gender (female vs. male)	0.857 (0.603 to 1.219)	0.391	0.981 (0.693 to 1.390)	0.916				
Therapies		0.090		0.080				
(Surgery + radiotherapy vs. only surgery)	0.799 (0.492 to 1.296)	0.363	0.893 (0.557 to 1.432)	0.639				
(Surgery + chemotherapy vs. only surgery)	0.918(0.642 to 1.423)	0.825	1.225(0.847 to 1.770)	0.281				
(Surgery + radiochemotherapy vs. only surgery)	0.918 (1.036 to 2.550)	0.035	1.701 (1.087 to 2.662)	0.020				
Tumor size		0.062		0.025		0.045		0.021
3–5 cm vs. ≤3 cm	1.285 (0.860 to 1.921)	0.222	1.404 (0.948 to 2.077)	0.090	1.378 (0.915 to 2.075)	0.124	1.432 (0.964 to 2.130)	0.076
>5 cm vs. ≤3 cm	1.657 (1.082 to 2.539)	0.020	1.787 (1.176 to 2.716)	0.007	1.730 (1.124 to 2.664)	0.013	1.821 (1.193 to 2.779)	0.005
pTNM-stage (III vs. I + II)	2.087 (1.443 to 3.019)	<0.001	1.956 (1.376 to 2.780)	<0.001	1.876 (1.267 to 2.778)	0.002	1.689 (1.162 to 2.456)	0.006
MYC	1.415 (1.043 to 1.920)	0.026	1.397 (1.041 to 1.874)	0.026				
PDIA3	0.618 (0.450 to 0.848)	0.003	0.638 (0.471 to 0.864)	0.004				
ITGA5B1	1.651 (1.216 to 2.241)	0.001	1.477 (1.098 to 1.986)	0.010				
Molecular prognostic model		<0.001		<0.001		0.001		0.006
Medium-risk vs. ≤ low-risk	1.830 (1.215 to 2.758)	0.004	1.625 (1.111 to 2.378)	0.012	1.577 (1.036 to 2.402)	0.034	1.493 (1.010 to 2.208)	0.045
High-risk vs. ≤ low-risk	2.914 (1.828 to 4.680)	<0.001	2.457 (1.580 to 3.823)	<0.001	2.539 (1.556 to 4.141)	<0.001	2.122 (1.338 to 3.367)	0.001

*Note.* Multivariate analysis, Cox proportional hazards regression model. Variables were adopted for their prognostic significance by univariate analysis.

## Data Availability

The clinical data and protein expression used to support the findings of this study are available from the corresponding author upon request.

## Supplementary Materials

Supplementary Materials Figure S1: representative images showing the scoring process by the automated quantitative pathology imaging system. Figure S2: predictive value of three genes and the molecular model in validation dataset. Table S1: the clinicopathological characteristics of validation dataset of patients with ESCC.
