# Supplementary material for: A Novel Three-Gene Model Predicts Prognosis and Therapeutic Sensitivity in Esophageal Squamous Cell Carcinoma
Source: Biomed Res Int. 2019 Nov 25;2019:9828637. doi: 10.1155/2019/9828637 (PMC6899311; doi:10.1155/2019/9828637)
Supplement: Supplementary Materials — Figure S1: representative images showing the scoring process by the automated quantitative pathology imaging system. Figure S2: predictive value of three genes and the molecular model in validation dataset. Table S1: the clinicopathological characteristics of validation dataset of patients with ESCC. [file 9828637.f1.docx]

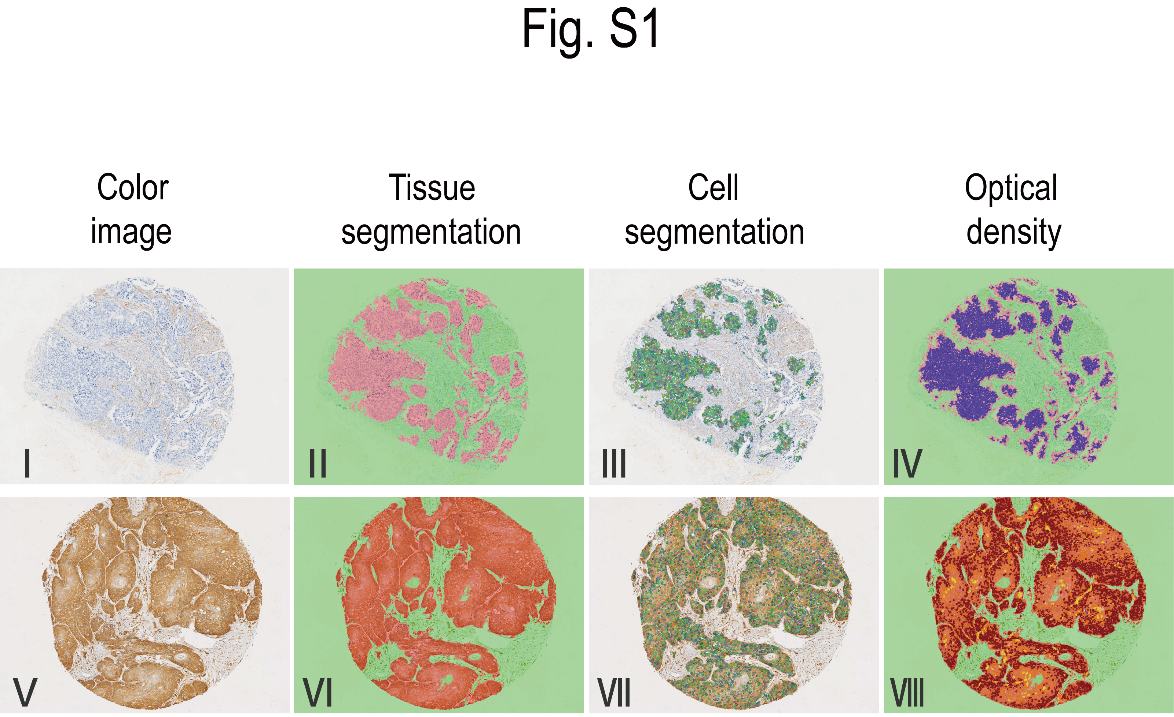


Figure S1. Representative images of the scoring process. (I/V) Color images of ESCC samples by IHC staining. (II/VI) Tissue segmentation of the color images. Red: tumor tissue; green: other. (III/VII) Cell segmentation of the tumor region. Green is representative of the cell nucleus, and the cytoplasm for each cell is shown in color. (IV, VIII) The cells are grouped into 4 tiers according to spectral analysis (Blue: 0, Yellow: +, Orange: ++, and Brown: +++). I-IV and V-VIII are from the same ESCC sample, severally.


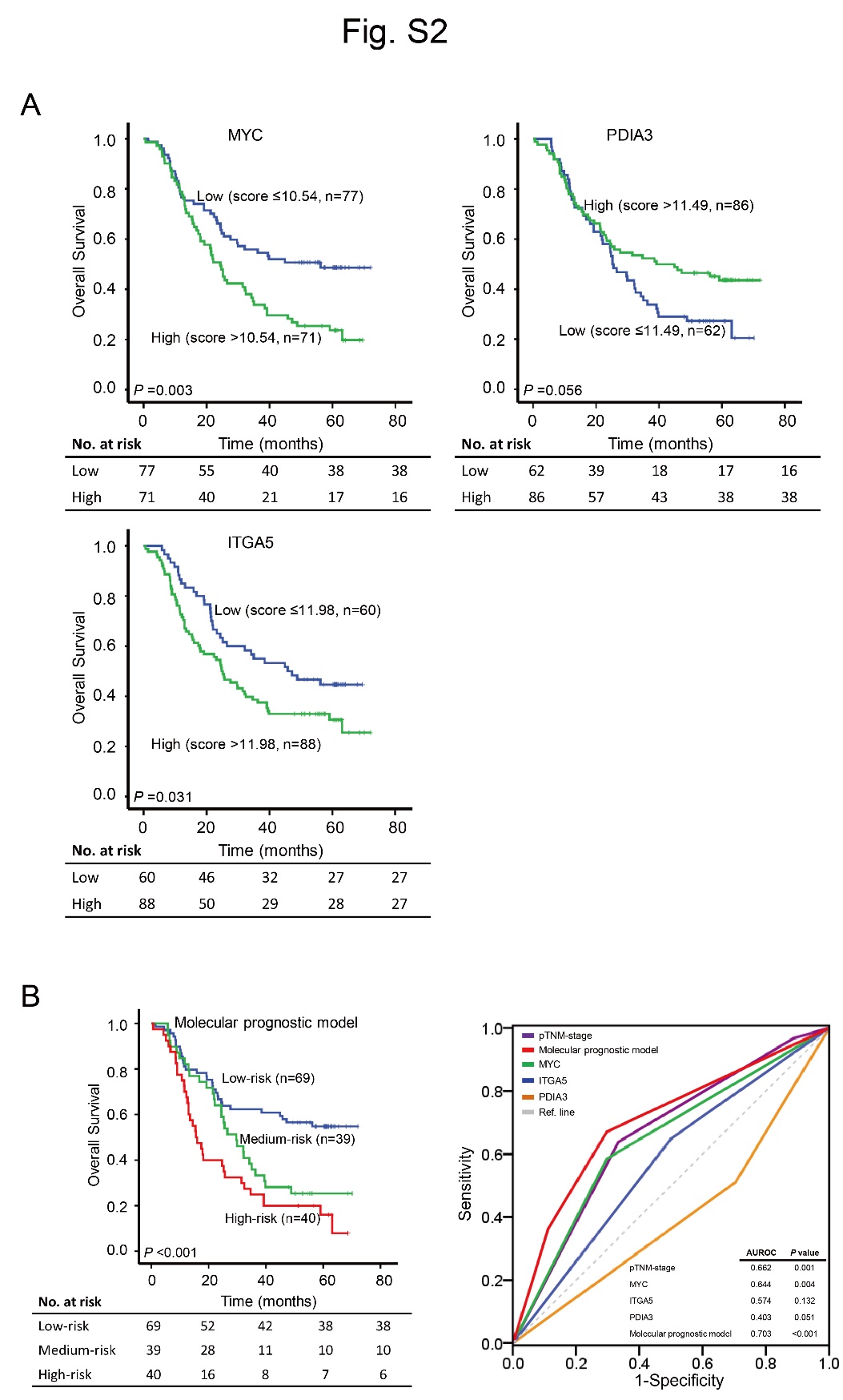


Figure S2. Predictive value of three-genes and the molecular model in validation dataset. (A) K-M survival analysis of OS based on the expression of MYC, PDIA3 and ITGA5. (B) K-M survival analysis and Receiver operating characteristic (ROC) curve was used to evaluate the prognostic value of the molecular model.

| **Table S1. The** **clinicopathological characteristics of validation dataset of patients with ESCC** | | | |
| --- | --- | --- | --- |
| **Clinical and pathological indexes** | **Case No.** | 5-year OS (%) | *P** |
| Specimens | 148 |  |  |
| Mean age | 59 |  |  |
| Age (year) |  |  |  |
| ≤59 | 72 | 40.5 | 0.139 |
| >59 | 76 | 32.9 |  |
| Gender |  |  |  |
| Male | 118 | 38.5 | 0.333 |
| Female | 30 | 27.8 |  |
| Tumor location |  |  |  |
| upper | 16 | 20.8 | 0.555 |
| middle | 80 | 34.6 |  |
| lower | 52 | 44.2 |  |
| Histologic grade |  |  |  |
| G1 | 28 | 39.8 | 0.296 |
| G2 | 83 | 39.7 |  |
| G3 | 37 | 25.5 |  |
| Invasive depth |  |  |  |
| T1 | 11 | 36.4 | 0.392 |
| T2 | 22 | 39.0 |  |
| T3 | 88 | 38.6 |  |
| T4 | 27 | 25.9 |  |
| Lymph node metastasis |  |  |  |
| N0 | 67 | 51.3 | 0.002 |
| N1 | 52 | 22.5 |  |
| N2 | 19 | 31.6 |  |
| N3 | 10 | 20.0 |  |
| pTNM-stage |  |  |  |
| I | 9 | 51.9 | <0.001 |
| II | 61 | 48.6 |  |
| III | 78 | 24.4 |  |
| *Log-rank test of Kaplan Meier method; *P* <0.05 was considered significant. | | | |
| All patients underwent surgical treatment. | | | |
| OS: overall survival | | | |
